# Kentucky State Dental Society

**Published:** 1874-07

**Authors:** 


### KENTUCKY STATE DENTAL SOCIETY.

The annual meeting of this society was held in the city of Louisville, on the 2d., and 3d., of June. About the usual number of members were in attendance. The meeting in many respects was a good one, especialy so far as the discussion of practical subjects was concerned.

There was quite a spirited discussion upon the relative merits of cohesive and non-cohessive gold foil for filling teeth; the chief advantages of each were pretty fully presented, and we doubt not the advocates of each received some new ideas.

There seems to be in the minds of some, who have uniformly adhered to the use of soft or non-cohesive to condemn strongly the use of co-hesive foil, some affirming that the latter is not fit to be used under any circumstances for filling teeth. The statement ought to be well sustained, when, as is the case, it is made in full view of the fact, that a very large majority of the profession, use cohesive foil largely and many exclusively. There are many fine operators with foil of each kind, and quite enough of inferior ones with anything.

There was some diversity of opinion in reference to the policy and government of the society. But such things will sometimes spring up in the best regulated institutions. This society has a charter from the State Legislature, impowering it to grant diplomas, and legally do whatever pertains to such an organization. This gives it a status and an importance, that should be highly appreciated by the dental profession of Kentucky.

We hope for a bright and efficient future for the Kentucky State Dental Society.

